# Clinical features and outcomes of *JAK2* unmutated erythrocytosis

**DOI:** 10.1007/s44313-025-00072-8

**Published:** 2025-03-31

**Authors:** Jeong Suk Koh, Wonhyoung Seo, Sora Kang, Myung-Won Lee, Ik-Chan Song, Deog-Yeon Jo

**Affiliations:** Division of Hematology/Oncology, Department of Internal Medicine, Chungnam National University College of Medicine, 282 Munhwa-Ro, Jung-Gu, Daejeon, 35015 Korea

**To the Editor:**

Polycythemia vera (PV) is a myeloproliferative neoplasm characterized by increased production of red blood cells independent of the mechanism that normally regulates erythropoiesis, which is associated with *JAK2* mutations [1]. Thrombotic and hemorrhagic vascular events are the main clinical manifestations of this disorder [2]. Established treatment guidelines for PV exist to reduce thrombotic vascular events [3, 4]. However, treatment strategies for *JAK2* unmutated erythrocytosis (hereafter ‘erythrocytosis’) vary due to the inconsistent risk of thrombosis [5, 6]. The clinical implications of erythrocytosis in the Korean population have rarely been described. Thus, we compared the clinical features and outcomes of *JAK2* unmutated erythrocytosis in comparison with those of PV in a Korean population.

We conducted a retrospective analysis of patients diagnosed with PV or erythrocytosis at the Chungnam National University Hospital between January 2010 and December 2022. PV was diagnosed according to the 2016 World Health Organization criteria [1]. In patients with PV, phlebotomy and hydroxyurea were prescribed according to the standard recommendations. Low-dose aspirin (100 mg/day) was prescribed to prevent thrombosis. *JAK2* unmutated erythrocytosis was defined as elevated hemoglobin or hematocrit levels fulfilling the PV criteria without *JAK2*V617F and *JAK2* exon 12 mutations. Phlebotomy was performed selectively in some erythrocytosis patients with a hematocrit greater than 50% and high cardiovascular risk. Thrombotic events included cerebrovascular accidents (ischemic stroke, transient ischemic attack, and venous sinus thrombosis), coronary events (ischemic heart diseases such as acute coronary syndrome), and splanchnic and peripheral thromboembolism. Events that occurred before, at, or after diagnosis were included in the analysis.

Descriptive data are presented as means ± standard deviations, medians (ranges), or percentages and were analyzed using Student’s t test, chi-square test, or Fisher’s exact test as appropriate. Correlations between hematocrit and other parameters were assessed using Pearson correlation analysis. The cumulative incidence of thrombosis was calculated using the Fine and Gray model, with death serving as a competing risk, and analyzed using the Gray equality test. Risk factors for thrombosis were also analyzed using the Fine and Gray regression model, with death serving as a competing risk. Overall survival (OS) was defined as the time from diagnosis to death due to any cause. Survival was estimated using the Kaplan–Meier method and analyzed using the log-rank test. Statistical analyses were performed using SPSS (ver. 24.0; IBM, Armonk, NY, USA) or SAS Studio (SAS Institute, Cary, NC, USA). In all analyses, *P* < 0.05 was taken to indicate statistical significance. This study was approved by the Institutional Review Board of the Chungnam National University Hospital (IRB No. 2022–11-070). The need for informed consent was waived by the ethics committee due to the retrospective study design.

A total of 282 patients were enrolled, 194 of whom had erythrocytosis and 88 had PV. Patients with erythrocytosis were younger (52 [17–84] years *vs.* 66.5 [31–91] years; *P* = 0.026), predominantly male (90.7% *vs.* 45.5%; *P* < 0.001), and more likely to have been diagnosed in the past six years (51.4% *vs.* 91.2%) (Fig. 1). Furthermore, erythrocytosis patients exhibited lower hematocrit levels (54.0 ± 3.4% *vs.* 46.7 ± 7.1%, *P* < 0.001), white blood cell counts (7.7 ± 2.8 × 10^9^/L *vs.* 15.5 × 10^9^/L, *P* < 0.001), neutrophil-to-lymphocyte ratios (2.3 ± 1.3 *vs.* 7.6 ± 6.7, *P* < 0.01), platelet counts (129.2 ± 53.5 × 10^9^/L *vs.* 532.4 ± 285.3 × 10^9^/L, *P* < 0.001), and normalized lactate dehydrogenase (LDH) ratios (0.9 ± 0.3 *vs.* 1.4 ± 0.5, *P* < 0.001) compared with PV patients. Conversely, erythrocytosis patients exhibited higher transferrin saturation (37.9 ± 35.8% *vs.* 13.9 ± 12.5%, *P* < 0.001), serum ferritin levels (201.1 ± 189.2 ng/mL *vs.* 41.8 ± 63.0 ng/mL, *P* < 0.001), and serum erythropoietin levels (12.5 ± 14.1 mIU/mL *vs.* 4.1 ± 5.3 mIU/mL, *P* < 0.001) compared with PV patients. Hypertension (47.9% *vs.* 28.9%, *P* = 0.004), sodium-glucose co-transporter-2 inhibitor use (12.9% *vs*. 0%, *P* < 0.001), smoking (37.1% *vs.* 21.7%, *P* = 0.003), chronic obstructive pulmonary disease (COPD) (8.8% *vs.* 2.3%, *P* = 0.044), heart failure (6.7% *vs.* 0%, *P* = 0.013), and elevated body mass index (> 30 kg/m^2^; 18.0% *vs.* 2.3%, *P* < 0.001) were more prevalent in erythrocytosis patients than in PV patients (Table 1). Hematocrit levels were positively correlated with neutrophil counts (*r* = 0.199, *P* = 0.005), neutrophil-to-lymphocyte ratios (*r* = 0.259, *P* < 0.001), serum LDH levels (*r* = 0.256, *P* = 0.001), serum iron levels (*r* = 0.183, *P* = 0.020), serum erythropoietin levels (*r* = 0.253, *P* < 0.001), and serum vitamin B_12_ levels (*r* = 0.226, *P* = 0.007) (data not shown). Of the 194 patients with erythrocytosis, 21 underwent a bone marrow study, and panmyelosis was not observed in any of these patients. The mean follow-up duration was significantly longer in PV patients than in erythrocytosis patients (42.5 [0.3–140.5] months vs 18.6 [0.1–137.9] months; *P* < 0.001). The overall cumulative incidence of thrombosis was significantly higher in patients with PV than in those with erythrocytosis (5-year incidence: 43.0% vs. 14.7%; 10-year incidence: 51.2% vs. 14.7%; *P* < 0.001). Although the cumulative incidence of thrombosis after diagnosis was higher in patients with PV than in those with erythrocytosis (5-year incidence, 12.7% *vs.* 5.3%, respectively), the difference was not statistically significant (*P* = 0.122). The cumulative incidence of thrombosis in patients with erythrocytosis was lower in the phlebotomized group than in the non-phlebotomized group (5-year incidence, 0% vs. 6.8%); however, the difference was not statistically significant (*P* = 0.280) (Fig. 2). Regression analysis identified diabetes mellitus (hazard ratio 4.93, 95% confidence interval, 1.26 − 19.31; *P* = 0.022) and COPD (hazard ratio 11.88, 95% confidence interval, 2.59 − 54.55; *P* = 0.001) as independent risk factors of thrombotic events in patients with erythrocytosis (Table 2). The overall survival tended to be lower in patients with PV than in those with erythrocytosis (5-year overall survival, 88.2% *vs.* 95.4%; 10-year overall survival, 65.8% *vs.* 95.4%, *P* = 0.064) (data not shown).

**Fig. 1:**
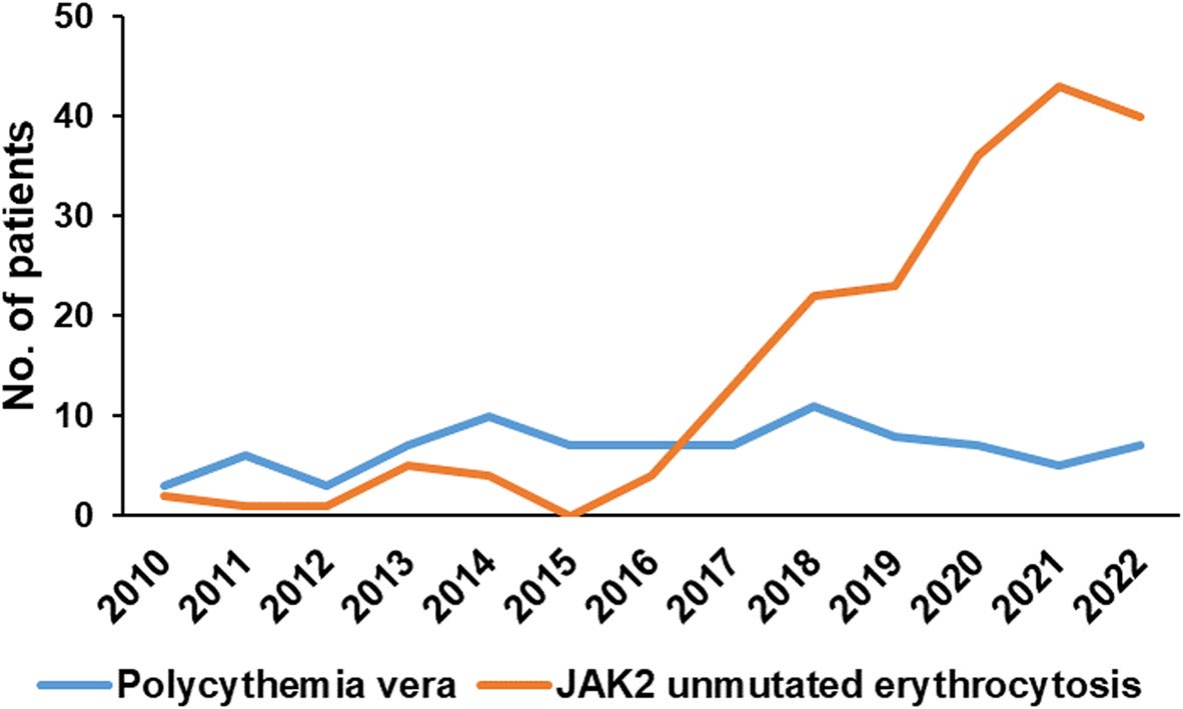
Changes by year in the number of patients newly diagnosed with polycythemia vera and *JAK2* unmutated erythrocytosis

**Table 1:** Patient characteristics

	Polycythemia vera (*N* = 88)	*JAK2* unmutated erythrocytosis (*N* = 194)	*P*
Age, yr, median (range)	66.5 (31 − 91)	52 (17 − 84)	0.026
Male, N (%)	40 (45.5)	176 (90.7)	< 0.001
Palpable splenomegaly, N (%)	3 (3.4)	0 (0.0)	0.030
Laboratory findings			
WBC (× 10^9^/L)	15.5 ± 6.5	7.7 ± 2.8	< 0.001
Neutrophil/Lymphocyte	7.6 ± 6.7	2.3 ± 1.3	< 0.001
Monocyte (× 10^9^/L)	0.7 ± 0.4	05 ± 0.2	< 0.001
Hemoglobin (g/dL)	18.1 ± 2.5	18.1 ± 1.1	0.784
Hematocrit (%)	56.7 ± 7.1	54.0 ± 3.4	< 0.001
Platelet (× 10^9^/L)	532.4. ± 285.3	129.2 ± 53.5	< 0.001
LDH (× ULN)	1.4 ± 0.5	0.9 ± 0.3	< 0.001
Transferrin saturation (%)	13.9 ± 12.5	37.9 ± 35.8	< 0.001
Serum ferritin (ng/mL)	41.8 ± 63.0	201.1 ± 189.2	< 0.001
Serum EPO (mIU/mL)	4.1 ± 5.3	12.5 ± 14.1	< 0.001
Driver gene mutation, N (%)			
*JAK2*V617F	84 (95.5)	0 (0.0)	
*JAK2* exon 12 mutation	3 (3.4)	0 (0.0)	
None	1 (1.1)	0 (0.0)	
Comorbidity, N (%)			
Hypertension	26 (28.9)	93 (47.9)	0.004
Diabetes mellitus	58 (65.9)	123 (63.4)	0.789
SGLT-2 inhibitor treatment	0 (0.0)	25 (12.9%)	< 0.001
Chronic kidney disease	20 (22.7)	50 (25.8)	0.583
Kidney transplantation	0 (0.0)	7 (3.6)	0.071
Current smoking	20 (21.7)	72 (37.1)	0.003
COPD	2 (2.3)	17 (8.8)	0.044
Sleep apnea	0 (0.0)	2 (1.0)	0.339
CHF	0 (0.0)	13 (6.7)	0.013
Dyslipidemia	22 (25.0)	64 (33.0)	0.177
BMI ≥ 30	2 (2.3)	35 (18.0)	< 0.001
Malignancy	4 (4.5)	6 (3.1)	0.541
Treatments, N (%)			
Cytoreductive treatment	74 (84.1)	0 (0.0)	< 0.001
Phlebotomy	81 (92.0)	28 (14.4)	< 0.001
Anti-platelet therapy	87 (98.9)	22 (11.3)	< 0.001
Anticoagulation	0 (0.0)	15 (7.7)	0.007
FU (mo), median (range)	42.5 (0.3 − 140.5)	18.6 (0.1 − 137.9)	< 0.001

*Abbreviations*: *LDH* lactate dehydrogenase, *ULN* upper limit of normal, *EPO* erythropoietin, *SGLT2* sodium-glucose co-transporter-2, *COPD* chronic obstructive pulmonary disease, *CHF* congestive heart failure, *BMI* body mass index, *FU* follow-up

**Fig. 2:**
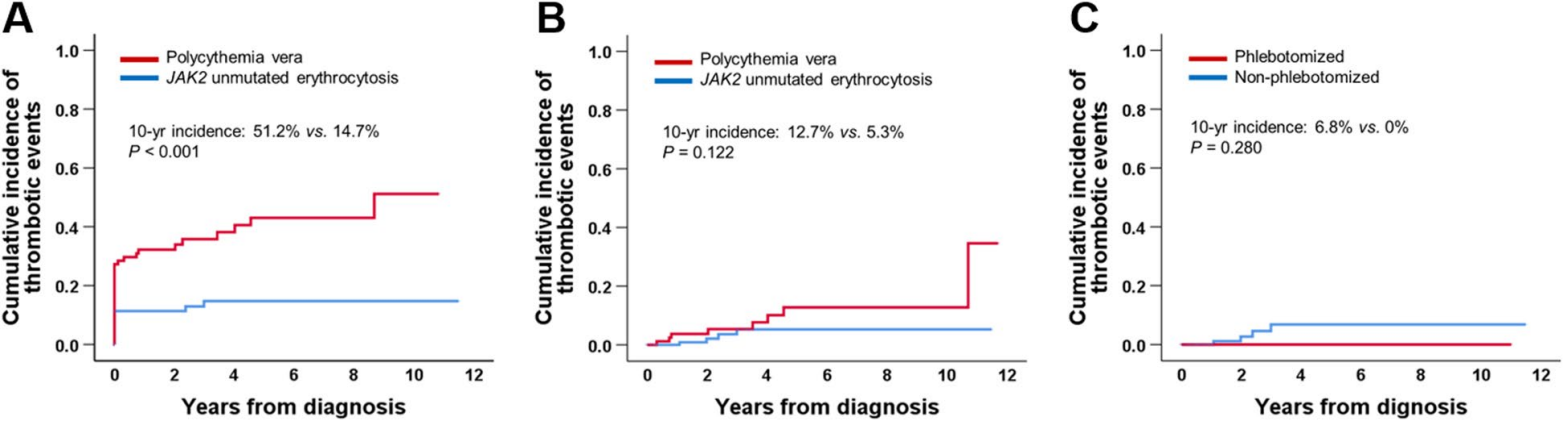
Overall cumulative incidence of thrombotic vascular events (**A**), cumulative incidence of thrombotic vascular events occurring after diagnosis (**B**), and cumulative incidence of thrombotic vascular events in patients with *JAK2* unmutaed erythrocytosis according to phlebotomy (**C**)

**Table 2:** Fine and Gray regression analysis of the risk factors for thrombotic vascular events in patients with *JAK2* unmutated erythrocytosis

	Univariate analysis			Multivariate analysis		
	HR	95% CI	*P*	HR	95% CI	*P*
Age > 60 yrs	4.17	1.78 − 9.75	0.001	2.67	0.64–11.21	0.179
Male	0.74	0.22 − 2.48	0.622	−	−	−
WBC > 12.0 × 10^9^/L	0.72	0.10 − 5.35	0.750	−	−	−
Neutrophil/Lymphocyte > 4	0.70	0.10–5.19	0.728	−	−	−
Monocyte > 1.0 × 10^9^/L	4.95	1.46–16.72	0.010	3.22	0.62–16.63	0.164
Hematocrit > 60%	2.13	0.50–9.04	0.307	−	−	−
LDH > 1.5 × ULN	4.57	0.62–33.99	0.138	−	−	−
Transferrin saturation < 20%	3.61	1.22–10.67	0.020	1.10	0.32–3.87	0.879
Serum ferritin > 150 ng/mL	0.10	0.01–0.76	0.026	0.07	0.01–0.56	0.012
Serum EPO > 19.5 mIU/mL	0.79	0.18–3.99	0.747	−	−	−
Hypertension	1.25	0.56–2.81	0.597	−	−	−
Diabetes mellitus	2.87	1.26–6.55	0.012	4.93	1.26–19.31	0.022
SGLT-2 inhibitor	2.26	0.90–5.68	0.084	−	−	−
Chronic kidney disease	1.65	0.72–3.79	0.234	−	−	−
Smoking	1.07	0.39–2.94	0.899	−	−	−
COPD	3.60	1.43–9.07	0.007	11.88	2.59–54.55	0.001
CHF	1.28	0.30–5.45	0.737	−	−	−
Dyslipidemia	2.03	0.91–4.53	0.082	−	−	−
BMI ≥ 30	0.97	0.33–2.84	0.954	−	−	−
Concurrent malignancy	1.27	0.17–9.40	0.817	−	−	−
Phlebotomy	1.11	0.38–2.35	0.855	−	−	−

*Abbreviations*: *HR* hazard ratio, *WBC* white blood cell, *LHD* lactate hydrogenase, *ULN* upper limit of normal, *EPO* erythropoietin, *SGLT-2* sodium-glucose co-transporter-2, *COPD* chronic obstructive pulmonary disease, *CHF* congestive heart failure, *BMI* body mass index

The diagnostic criteria for PV have been continuously revised [1, 7–10]. Notably, the diagnostic thresholds of hemoglobin and hematocrit for PV have been considerably lowered in the diagnostic criteria proposed by the World Health Organization. The number of newly diagnosed patients with PV is increasing in Korea [11], which is attributable, at least in part, to changes in diagnostic criteria and widespread studies of driver gene mutations. In parallel, the number of *JAK2* unmutated erythrocytosis cases is increasing possibly for reasons similar to those of PV. The present study showed that although *JAK2* unmutated erythrocytosis carries a significantly lower thrombotic risk than PV, individuals with erythrocytosis, particularly those with diabetes mellitus and COPD, have a persistent risk of thrombotic vascular events. Further studies are warranted to determine whether thrombophylaxis is required in these patients. The therapeutic benefits of phlebotomy in these patients also require further investigation.

## Supplementary Information

Supplementary Material 1.

## Data Availability

No datasets were generated or analysed during the current study.
