# Supplementary material for: Clinical features and outcomes of JAK2 unmutated erythrocytosis
Source: Blood Res. 2025 Mar 31;60(1):20. doi: 10.1007/s44313-025-00072-8 (PMC11958889; doi:10.1007/s44313-025-00072-8)
Supplement: Supplementary file 1 — Supplementary Material 1. [file 44313_2025_72_MOESM1_ESM.docx]

**Supplementary** Table 1. Thromboembolic and hemorrhagic vascular events.

|  | Polycythemia vera (N=88) | *JAK2* unmutated erythrocytosis (N=194) |
| --- | --- | --- |
| Initial event, N (%) | | |
| Cerebrovascular Stroke | 18 (20.5) | 9 (4.6) |
| TIA | 0 (0.0) | 0 (0.0) |
| VST | 0 (0.0) | 0 (0.0) |
| Any | 18 (20.5) | 9 (4.6) |
| Coronary ACS | 5 (5.6) | 6 (3.1) |
| Others | 7 (8.0) | 7 (3.6) |
| Any | 12 (13.6) | 13 (6.7) |
| Splanchnic Arterial | 0 (0.0) | 1 (0.5) |
| Venous | 1 (1.1) | 1 (0.5) |
| Any | 1 (1.1) | 2 (1.0) |
| Peripheral Arterial | 1 (1.1) | 1 (0.5) |
| Venous | 1 (1.1) | 0 (0.0) |
| Any | 2 (2.2) | 1 (0.5) |
| Total | 33 (37.5) | 25 (12.9) |
| Involved vessels of the initial events N (%) | | |
| Arterial | 31 (35.2) | 24 (12.4) |
| Venous | 2 (2.3) | 1 (0.5) |
| Any | 33 (37.5) | 25 (12.9) |
| Pooled events, N | | |
| Type of events | | |
| Cerebrovascular Stroke | 18 | 9 |
| TIA | 0 | 0 |
| VST | 0 | 0 |
| Coronary ACS | 6 | 6 |
| Others | 9 | 9 |
| Splanchnic Arterial | 0 | 1 |
| Venous | 1 | 1 |
| Peripheral Arterial | 1 | 1 |
| Venous | 1 | 0 |
| Total | 36 | 27 |
| Timing of the pooled events, N (%) | | |
| Before diagnosis | 18 (50.0) | 19 (70.4) |
| At diagnosis | 10 (27.8) | 4 (14.8) |
| After diagnosis | 8 (22.2) | 4 (14.8) |
| Subtotal | 36 (100) | 27 (100) |

Abbreviations: TIA, transient ischemic attack; VST, venous sinus thrombosis; ACS, acute coronary syndrome.
